# Experimental generation of fulgurite under realistic lightning discharge conditions

**DOI:** 10.1038/s41598-023-38781-8

**Published:** 2023-07-19

**Authors:** A. Zeynep Çalışkanoğlu, Alessandra S. B. Camara, Corrado Cimarelli, Donald B. Dingwell, Kai-Uwe Hess

**Affiliations:** 1 Department of Earth and Environmental Sciences, Ludwig-Maximilians-Universität, Theresienstraße 41, 80333 Munich, Germany; 2 Institute of Energy Systems Munich, Universität der Bundeswehr, Werner-Heisenberg-Weg 39, 85577 Neubiberg, Germany

**Keywords:** Geology, Mineralogy, Volcanology, Materials science, Physics

## Abstract

Fulgurites have been documented in geological deposits from throughout Earth's history. They have also been assigned a potential role in prebiotic chemistry as a source of reactants. Fulgurites are generated in nature by cloud-to-ground lightning strikes. The unpredictability in space and time of the occurrence of lightning events has limited the investigation of both the mechanisms by, and the conditions under, which fulgurites form. A laboratory-based approach can mitigate these limitations. Here, we describe experimentally generated fulgurites generated from Laacher See volcanic ash. We employ a DC source with a trigger-pulse setup in a high voltage laboratory, whose capabilities enable experimental conditions that correspond closely to the electrical characteristics of natural lightning strikes. The experimentally generated fulgurites closely resemble naturally-occurring fulgurites in both state and texture. These experimental investigations yield a high reproducibility of the characteristic of fulgurites generated under well-constrained conditions, enabling some inferences to be made regarding the processes involved in the generation of fulgurites in nature. This work provides a basis for a systematic characterization of experimental fulgurites and the characteristic of lightning discharges.

## Introduction

Fulgurites, from the Latin word for lightning—*fulgur*, generally take the form of natural glassy irregular tubes generated by lightning strikes in the sand, soil, or rock. Natural fulgurites were first described (in sandy sediments) by Herman in 1706^1^, and the oldest fulgurite found to date has been speculated, on the basis of the fossiliferous Carboniferous rocks overlying the host, to be of Permian age^2^. The fulgurites present on Earth form by cloud-to-ground lightning strikes (by either thunderstorms or volcanic eruptions) or as a result of accidents involving electric transmission lines^3–5^. Natural fulgurites have been explored in terms of their morphological and chemical states^6–9^, including the description of high-temperature mineralization^10^, reconstruction of paleoecology^11^, and evaluation of the availability of chemical sources for pre-biotic chemistry^12–14^.

Experimentally generated fulgurites have also been briefly described in some preliminary feasibility studies^15–20^. These valuable pioneering scientific inquiries have generally not been subject to the standard protocols of the natural lightning research community and generally lack the reproducibility and precision of a systematic approach. The reasons generally lie in the experimental technologies employed. The current generated by the Leyden jar battery (ca. 20–60 kV) was, for example, insufficient to create fulgurites closely resembling natural ones^15,16^. Subsequent experiment setups were missing inductivity^17^, and/or they produced solely a first-return stroke component (the component of the lightning discharge vital for breaking down the dielectric field strength of the original material)^18,21^. The pulse they generated using the sinusoidal waveform aids the dielectric breakdown of the sample, ultimately generating melting, but it is not sufficient to reproduce the typical morphology of fulgurites^19^. The produced current (50 A) by the electro melt simulator for 200–300 ms used by Castro et al.^20^ was well below the current condition of natural lightning (∼30 kA for negative polarity and ∼300 kA for positive polarity flashes^22^).

The experimental setup used in our study (DC source with trigger pulse) has been designed and constructed to ensure compliance with the lightning research community’s recommendations for lightning strike studies (e.g., waveforms IEC 62305^23^) and is located at the Universität der Bundeswehr (UniBw), Munich, Germany. An major advantage of this setup is that these experiments can be easily and very precisely reproduced.

Here, we use a volcanic ash from the Laacher See, East Eifel volcanic field, Germany as pristine material for the generation of the experimental fulgurites and systematically describe their formation mechanism and characteristics as a function of the varying conditions of the discharges.

## Natural fulgurites

**Figure 1:**
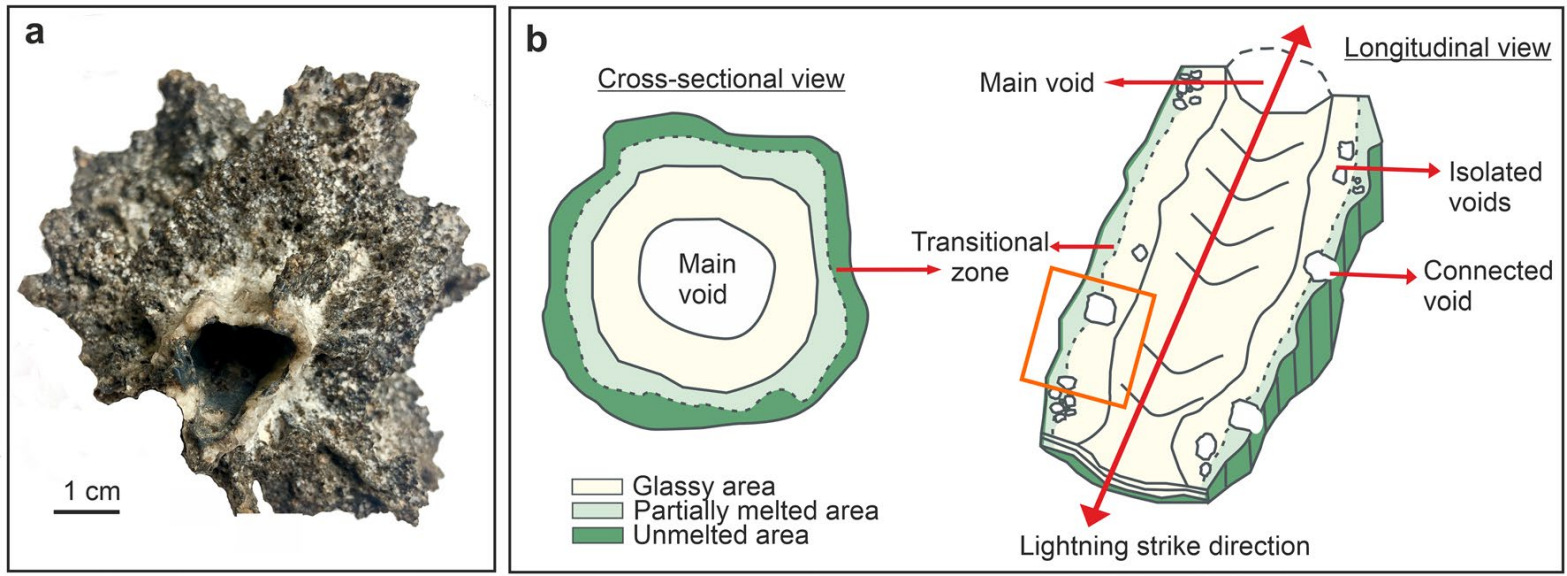
(**a**) An example of natural fulgurite (i.e., sand) from the Sahara Desert. (**b**) A representative cross-sectional and longitudinal views of a natural fulgurite with detailed morphological description. The orange square in the longitudinal view corresponds to the area where BSE images of the generated fulgurites in Fig. 4a–e are presented.

Here, we define some basic characteristics of natural fulgurites to facilitate the comparison of natural fulgurites with their experimental counterparts. Natural fulgurites may exhibit diverse compositions and colors, which depend on the pristine material chemistry, possible interactions and reactions, and chemical diffusion between phases in contact upon melting. Such fulgurites share some general morphological characteristics; commonly exhibiting sub-circular cross-sectional structures characterized by a central void (either open, partially-closed or closed) which represents the path of lightning propagation into the target material. Their lengths vary from centimeters to several meters, with variable wall thickness. The geometry of the central void can reveal tortuous morphologies with branching reflecting secondary conductive paths along the main direction of propagation of the lighting discharge. The external surfaces of natural fulgurites typically display a rough texture and may be partially covered with the remains of pristine material and partially melted crystals adhering to the melted fulguritic mass. In contrast, the fulgurites' interior surfaces generally exhibit a vitreous smooth surface surrounding the central void. A remainder of partially melted crystals can be found on the surface of the glassy volume of the fulgurite. A representative natural fulgurite sample from the Sahara Desert in Africa^24^ and a general cross-sectional and longitudinal view illustrating the fundamental morphological features of a fulgurite are presented in Fig. 1.

## Experimental setup

**Figure 2:**
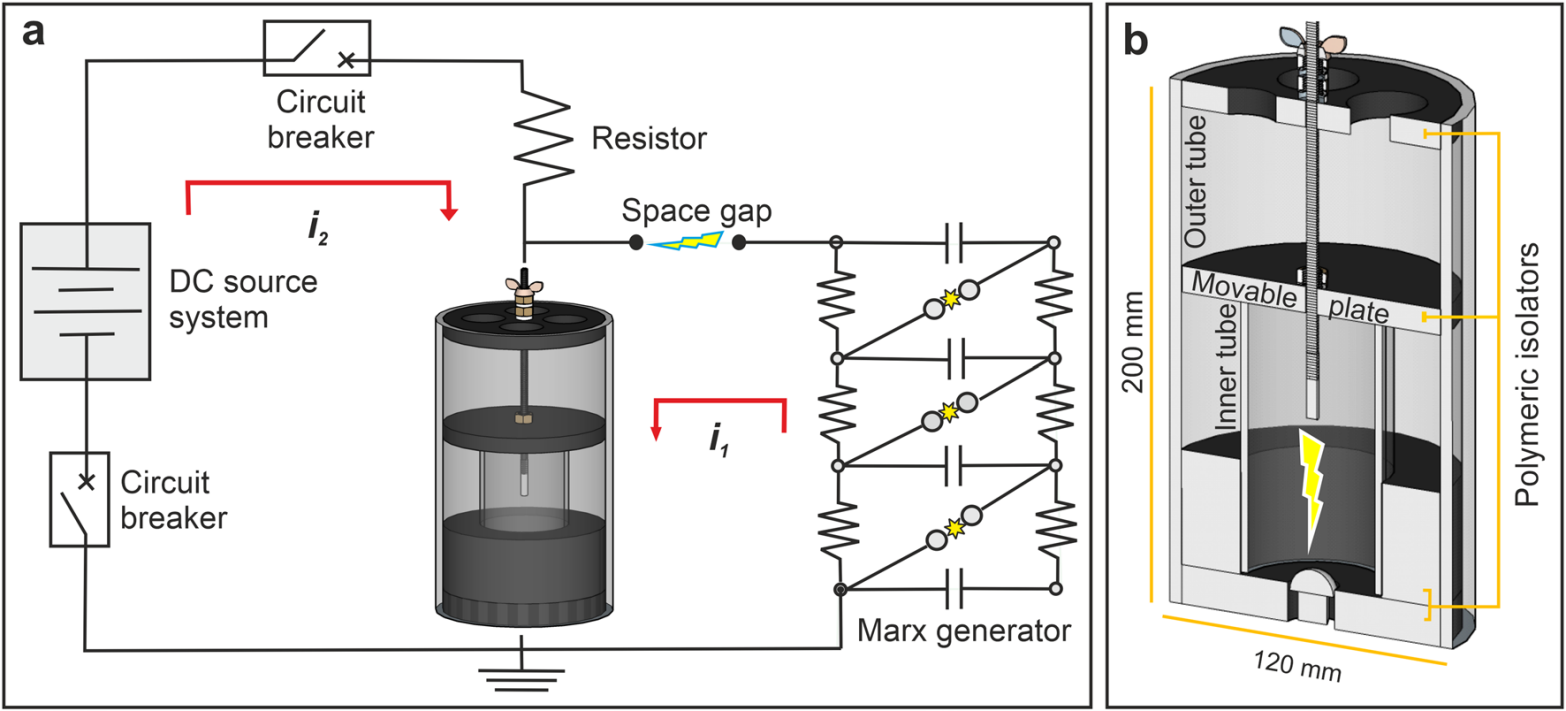
Schematic view of the experimental setup. (**a**) Circuit diagram of the DC source with trigger-pulse setup and 3D image of the sample container. The first return stroke (***i***_***1***_) indicates the lightning first return stroke component and the long-duration continuing current (***i***_***2***_) indicates the lightning continuous current component. (**b**) A sectional view of the cylindric sample container.

We simulated lightning discharge in an insulated sample container by a DC source with a trigger-pulse setup (Fig. 2a). The DC setup simulates the effects of natural lightning current discharges within controlled and highly reproducible conditions. The main elements of the setup are a Marx-generator, a battery system (DC source) consisting of 60 silver-lead-acid batteries of 12 V connected in series (720 V–600 A), circuit breakers and switches, a switching controller and an extender safety circuit (Fig. 2a). The setup is designed to represent the main current components of natural lightning^25^, namely: (1) first return stroke, which has the highest current amplitudes—typically of the order of 10^3^ A lasting up to several 100 µs; (2) subsequent return stroke, which is lower in amplitude and duration but often with a significantly higher current (up to a few 100 kA/µs); (3) the long-duration continuing current, which exhibits much lower current amplitudes of just a few 100’ A^26^, but of much longer duration (generally lasting up to 100 ms). In our setup, the first return stroke is initiated by a voltage impulse, using the Marx-generator (impulse currents of 1500–5000 A for approximately 100 µs) to trigger the spark gap between electrodes located in the insulated sample container, thus allowing the breakdown of the dielectric strength of the target material. Next, the DC source initiates the production of the long-duration continuing current. Two circuit breakers are arranged to stop this current at a pre-set time duration. In our experiments we simulate positive lightning discharges by positively charging the top electrode while the bottom one is negatively charged. Negative discharges can also be simulated with the same setup and produce fulgurites showing no significant difference from those generated by positive discharges. Fulgurite generation by positive discharges is however preferred for practical reasons during the preparation of the experiments. A video of one very similar experiment can be seen in Çalışkanoğlu et al.^14^.

The insulated sample container has two coaxial cylindrical containers made of 3 mm thick plexiglass tubes: the inner tube (height (h): 200 mm and diameter (D): 120 mm) contains the sample while the outer tube (h: 60 mm and D: 60 mm) prevents mobilization and dispersion of the sample in the laboratory space when hit by the discharge (Fig. 2b).

Electrically non-conductive and mechanically resistant polymeric cylindrical disc plates are located at three positions within the sample container (top, middle and bottom). The top and bottom plates are fixed, whereas the middle plate is designed to be movable, which enables the adjustment of the distance between electrodes to optimize experimental conditions and containing thereby the consequences of any abrupt mechanical shock in the container during the experiments. The lightning discharge is generated between two electrodes with different geometries: a bullet-shaped upper electrode (h: 27.42 mm and D: 13 mm) attached to the middle plate and a dome-shaped lower electrode (h: 19 mm and D: 46 mm) attached to the bottom plate.

All the experiments were conducted at atmospheric temperature and pressure. The environmental conditions in the laboratory were measured regularly by a GFTB 100 precision Thermo-Hygrometer and Barometer, which showed a stable environment (T: ~ 19.2 °C ± 0.2, P: ~ 946 hPa ± 10 and RHmean: ~ 50.0% ± 0.6) during the experiments.

**Figure 3:**
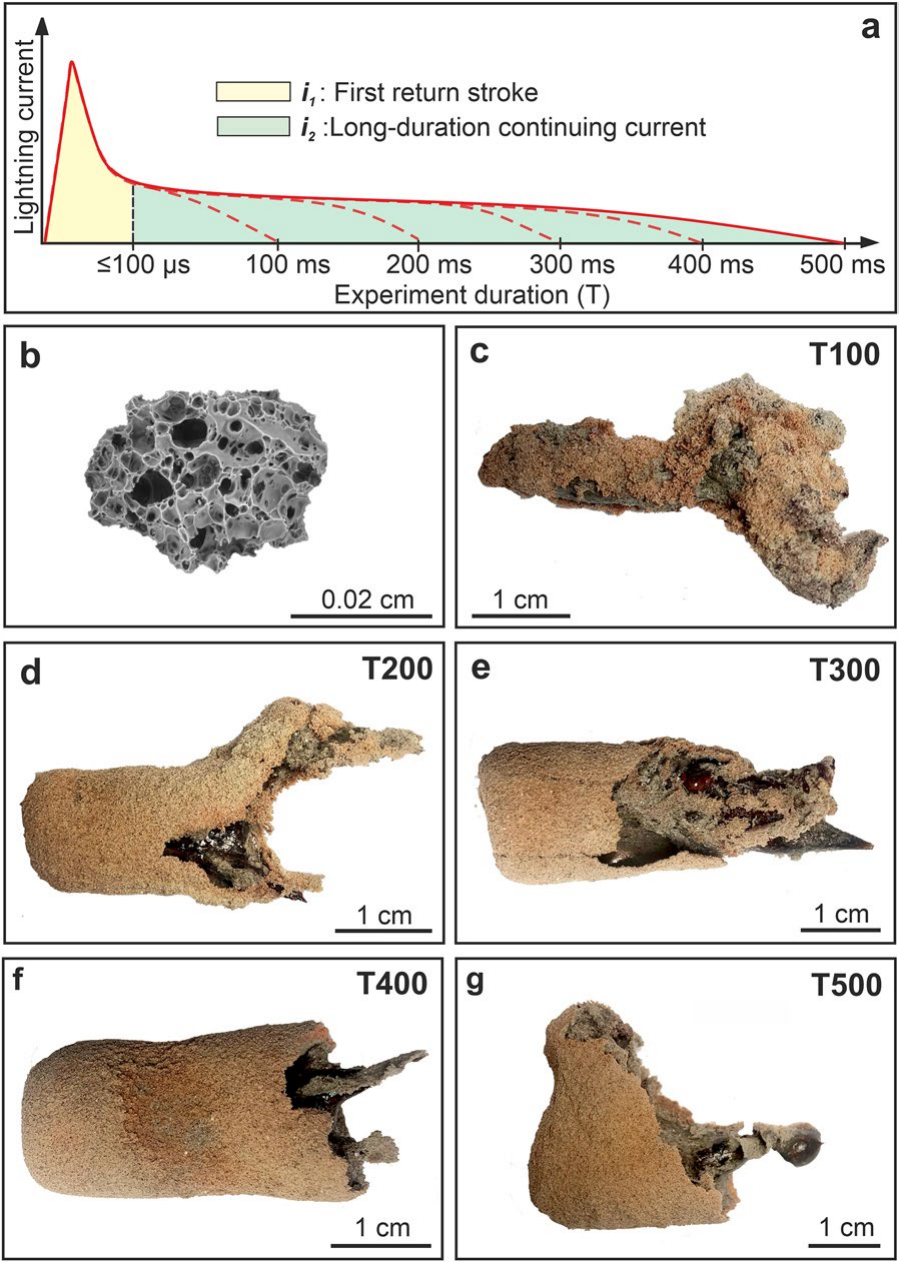
(**a**) Schematic composite electrical waveform of the experimental lightning components ***i***_***1***_ and ***i***_***2***_ for different experiment durations in µs and ms. (**b**). A SEM-BSE image of a pristine clast of the LSB. (**c**–**g**) Pictures of experimentally generated fulgurites under the variable long-duration continuing current (100–500 ms). The left end of each fulgurite corresponds to the portion adhering to the lower electrode in the experimental setup.

Three test experiments were performed to adjust the distance between the electrodes and to determine the optimal amount of target material to fill the inner tube. Subsequently, six experiments were performed under similar experimental conditions (***i***_***1***_ : up to a few 100 kA/µs and ***i***_***2***_ : 220 A–350 A) but with varying duration (0, 100, 200, 300, 400, and 500 ms). The lowest duration time of continuing current allowed by our setup is 100 ms which is also close to the average duration (nearly 150 ms) for long-duration continuing current produced by natural lightning as claimed by Brook et al.^27^. From 200 to 500 ms experiments were conducted to examine the duration of extremely prolonged continuing current effect on the fulgurites’ structure. Hereafter we will refer to the experiments as T0, T100, T200, etc. according to the characteristic duration of the continuing current phase. The T0 experiment was performed by striking the sample with the first return stroke alone with no continuing current phase. The inner tube was filled with 200 g of target material for each experiment and all experiments were performed with new sample batches. The spacing of the electrodes was held constant at 50 mm for all the experiments. It is worth noticing that the fixed distance between the electrodes largely determines the maximum length of the experimental fulgurites. A schematic electrical waveform generated by the setup for each experiment duration can be seen in Fig. 3a (current waveforms with digital oscilloscope: Supplementary Figs. S1).

## Target material

As pristine material for the experiments we used pre-sieved volcanic ash particles of Laacher See pumice, the “Laacher See Bimse” (hereafter LSB) deposits produced during the 13 ky eruption of Laacher See volcano (Eifel, Germany) provided by ROTEC GmbH. We use the fraction size of LSB particles between 90 and 300 µm with the addition of 3 wt% of very fine ash (< 63 µm)^28^. The LSB is a multi-phase material consisting mainly of vesiculated glass particles, lithic fragments and loose crystals^29^ which presents connected and isolated porosity (Fig. 3b). The juvenile material is phonolitic in composition (SiO_2_ ∼55 wt%; Al_2_O_3_ ∼20.5 wt%; NaO ∼11 wt%; K_2_O ∼5.5 wt%) and the main crystalline phases (5–8%) are quartz, plagioclase, sanidine, and clinopyroxenes^30,31^. The feldspar and quartz crystal grains are ∼1 wt% of the total bulk composition and the average fraction size of these crystals is 250 µm. The average density of the LSB used here was measured as 2.42 ± 0.02 g/cm^3^ by helium pycnometer^32^. The LSB volcanic ash was chosen as starting material as it has been extensively characterized and deployed in previous lightning discharge experiments^28,32–34^. The grain size distribution of LSB approximates that of silt and sand sediments where fulgurites are often found. Moreover, its mineral assemblage contains quartz and feldspars which are common minerals in sediments. Hence, this volcanic granular material offers the advantage of studying physical and chemical modification of its glass and crystal grain components upon lightning-induced melting reactions.

## Experimentally generated fulgurites

### Morphology

We generated 5 fulgurites in 6 experiments. In the T0 experiment (100 µs impulsive current and no continuing current) was only found some partial melting and sintering of particle clusters. On the contrary, all experiments characterized by variably long-continuing currents (T100 and T200, etc.) did generate fulgurites (Fig. 3c–g). The exterior surface of all fulgurites exhibit the reddish-brown color of the pristine material. Most of the surface of each fulgurite is covered by remnants of pristine material and partially melted crystals. The fulgurites from T100 to T400 experiments have a similar length of 45 mm, while the T500 fulgurite has a length of 35 mm, i.e., 5 and 15 mm shorter than the distance between the electrodes, respectively. The thicknesses of the unmelted and partially melted area of the fulgurites vary up to 0.6 mm and 0.5 mm, respectively, and the glass area exhibits variable thickness overall, regardless the experiment duration. The main void also exhibits a variable diameter in different parts of the fulgurite reflecting its irregular geometry. The average diameter of T100, T200, T300 and T500 fulgurites is around 25 mm, whereas T400 fulgurite is about 20 mm in diameter. All fulgurites exhibit a tube-like morphology, exception made for T500. The T100, T200, and T500 fulgurites display short apophyses (branches). An open central void is only observed in the T200 fulgurite. The total mass of the fulgurites (2.432 g—T100, 4.998 g—T200, 6.002 g—T300, 8.370 g—T400, and 9.589 g—T500) shows a gradual increment with increasing duration of the continuing current.

### Mineralogy

**Figure 4:**
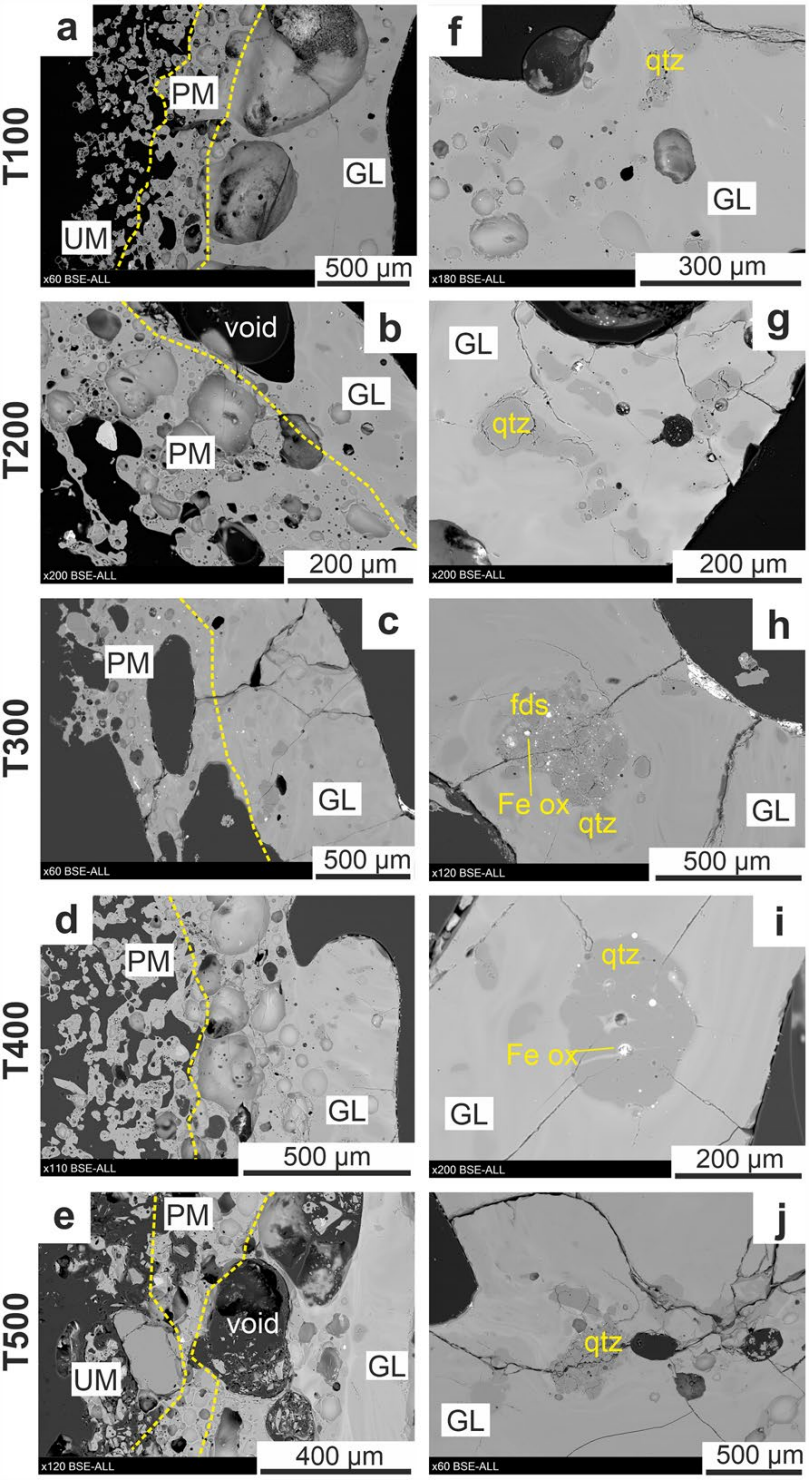
(**a**–**e**) BSE images of half face of each cut fulgurite as referred in Fig. 1b. Yellow dashed lines on the images mark the transitions between textural domains. (**f–j**) Images of the partially melted crystals in the glassy area of the fulgurites. *qtz* Quartz, *fds* Feldspar, *FeO* Iron-oxides, *GL* glassy area, *PM* partially melted area, *UM* unmelted area.

All fulgurites have been sectioned longitudinally (i.e., parallel to the direction of lightning discharge). Cross sections from the glassy area (center) to the external area are shown in Fig. 4a,e. Partially melted crystals are detected in partially melted and glassy area. They are mostly constituted by quartz with minor Fe-oxide and K-feldspar, while no residual pyroxene crystals were observed (Fig. 4 h,j). The other oxides (i.e., Cupper and Wolfram composites) are also found and their size (< 10^3^ nm) is tiny to present difficult to resolve in the BSE images. The size and the fraction of these crystals and oxides show no change with increasing experiment duration, nor do they exhibit a specific spatial distribution.

Voids are observed in all fulgurites. No correlation was found between their size, number and spatial distribution with the duration of each experiment. They display complex shape due to the presence of the partially melted crystals. Some of the voids also appear as coalesced. They predominantly cluster to form a layer between the glassy and partially melted areas, but some voids are also detected within the glassy areas. The voids in the glassy area are up to 20 mm in length, whereas the voids between the unmelted and partially melted areas are up to 0.7 mm in length. (Fig. 4c).

### Density and porosity

**Figure 5:**
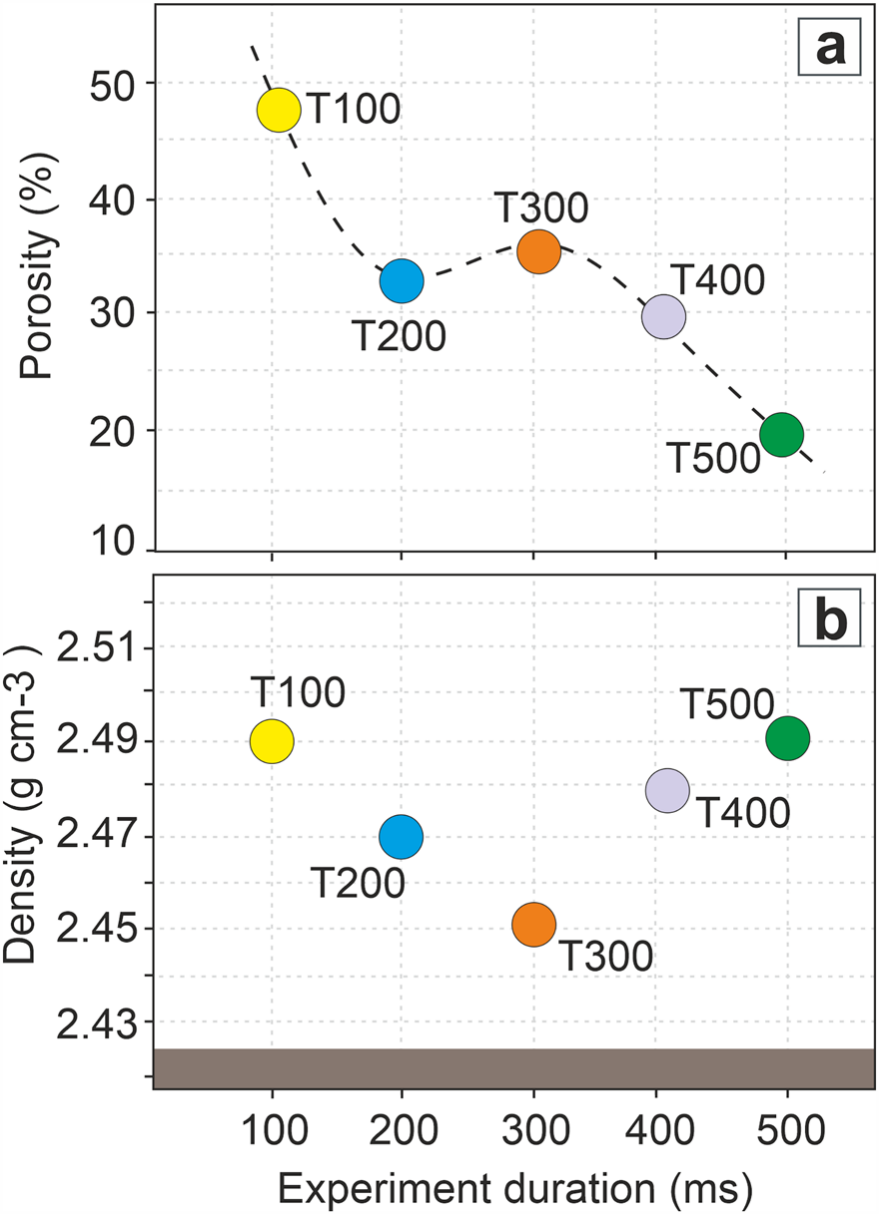
(**a**) The porosity calculation from 2D image analysis of all fulgurites. (**b**) Density measurements of LSB, synthesized LSB glass and all fulgurites. The brown area represents LSB (2.42 ± 0.02 g/cm^3^) and synthesized LSB glass (2.42 ± 0.004 g/cm^3^) densities for comparison. The error of the calculated and measured values for each experimental run is within the size of symbols.

In order to quantify the density and porosity as a function of melting duration, we used 2D image (BSE) analysis as representative of the 3D vesicularity combined with helium pycnometry data (Fig. 5a,b; Supplementary Table S1). The density of the pristine material (glass + multi-phase crystals) is 2.42 ± 0.02 g/cm^3^^32^. Firstly, all pristine material adhering to the solid fulgurite were cleaned by brushing before being crushed to ≤ 63 µm to for the correct evaluation of the analyses. The fulgurites (glass and partially melted crystals, possible minor unmelted crystals) exhibit density value around 2.48 ± 0.02 g/cm^3^. The T300 is the least dense among all fulgurites with around 2.46 ± 0.007 g/cm^3^. The density value (2.42 ± 0.004 g/cm^3^) of the synthesized LSB glass (glass and possible minor microcrystals) were also measured to compare with all fulgurite samples to see expansion ratio of fulgurites. The fulgurites indicate a slightly higher density than LSB and synthesized LSB glass.

A mosaic of BSE image of each fulgurite is used to estimate porosity ratio. To match the density measurement, we exclude the voids among the pristine particles in the unmelted area. It is found that the porosity ratio of the fulgurites (glassy + partially melted area) reduces from ∼47% in T100 to ∼18% in T500.

## Discussion

We successfully generated five fulgurites using natural volcanic ash samples of phonolitic composition and consistent grain size (< 300 µm) distribution by exposing the ash to high-current impulsive transients and variable duration of continuing current. Although our setup does not allow a direct observation of the formation of the fulgurites, videography of the experiments and a detailed characterization of the resulting fulgurites, together with the imposed experimental conditions, allow insight in the fulgurite formation. Our fulgurites present a tube-like shape with thick glass wall and are coated with thin pristine particles (crystals + glass) and partially melted crystals. These fulgurites do not fit into the fulgurite classification (Type I-sand, II-soil, III-caliche, VI-rock and V-droplet) proposed by Pasek et al.^7^. Although that classification scheme tackles the difficult task of ordering the complex nature of fulgurites, it does not include fulgurites from volcanic protoliths. Material composition as well as size and nature of its components -especially when the protolith consists of incoherent sediments or clastic rocks- play a crucial role on the morphology, density, porosity and glass proportion of the fulgurite considering the variations in the current flow and duration of the lightning strike.

In the high current and high voltage (up to a few 100 kA and 150 kV) experiment, the starting material was exposed to a high energetic event only for a very short time (100 µs) and some partial melting of particle clusters were found. Fulgurites were generated only in the high voltage and current impulse experiments with a continuing current (220 A–350 A) of variable duration (100–500 ms) (Fig. 3c–g). Results of the high current impulse experiments by Genareau et al.^21^ also supports our finding in that the initial high current impulse alone is not sufficient to produce enough melting to generate a fulgurite body. The addition of a prolonged continuing current phase, instead, allows the necessary energy (heat) transfer required to sustain the melting process of the pristine material and is often associated with lightning-ignited fires^35–37^. Measured continuing currents in natural lightning have revealed variable durations and have been referred to as very short (3–10 ms), short (10–40 ms) and long (> 40 ms) by Lapierre et al.^38^. Studies show that, although infrequent, continuing current following return strokes can last longer than 100 ms and can exceed 350 ms^38,39^. In our study, the minimum continuing current duration at which substantial melting is first observed is 100 ms (experiment T100). This is also the shortest continuing current duration that can be achieved with our setup; hence we cannot exclude that melting may be already produced by shorter continuing currents, other parameters (i.e., composition, grain size distribution and electrodes gap) kept constant. A striking difference in the structure of the fulgurite is observed between experiments with no continuing currents and continuing current of 100 ms (i.e., experiments T0 and T100, respectively). Exposure of the pristine material to longer continuing currents (i.e., T200 and T300) does not produce substantial structural and chemical changes of the experimental fulgurites relatively to T100.

The state of the pristine material and the lightning discharge characteristics have a strong influence on the formation process of the fulgurites. Silicate glasses are more suitable target materials for fulgurite generation due to the lower melting temperature of glass as compared to the melting temperatures of silicate minerals. Presence of organic matter (i.e., lichen, roots) in the pristine material can also create local positive electrostatic charge to which the lightning can attach^19^. Burnt organic matter might thus increase the opportunity to find elements in their reduced form (i.e., phosphorus) in the fulgurite composition^40,41^. These elements are also referred as a substantial matter for the organic forms^7^, which makes fulgurite attractive for emergence of life studies. Presence of organic matter in our pristine samples is to be excluded as the grains are obtained by fresh rock exposures. However, some level of chemical weathering cannot be excluded a priori.

Additional experiments by our group (not presented here) and Teixeira^42^, run under similar electrical conditions (constant experiment duration—500 ms), have also revealed that increasing the proportion of larger grains in the target material, plays a vital role on the fulgurite formation process, independently on the mineral phase composition. A prevalence of the larger grains (> 300 µm) in the pristine material seem to prevent the shaping of the fulgurite in its form, while they seem not to prevent the shaping of the fulgurite when in moderate to low amounts. However, as shown in this study, larger grains undergo thermal deformation from their outer boundaries (Fig. 4f–j). On the other side, Teixeira^42^ indicates that smaller grains (40–150 µm—quartz) were completely melted in the formation of the fulgurite. Wadsworth et al.^43^ also support our finding in that the edges of the smaller (nearly 310 µm) volcanic ash particles would round up in the ionized lightning channel for heating durations of 3 ms and temperature exceeding 3000 K, while larger grains would retain their original shape. Elmi et al.^40^ show that an uncrushed holocrystalline rock (an unaltered block of granitic rock sample) exposed to the AC source voltage (up to 150 kV) with 26.5 cm distance of electrodes did also not generate any melt. This indicates that after the grain size exceeds a certain limit formation of a fulguritic mass is greatly inhibited.

The morphology of the fulgurite is highly associated with the state of the pristine material. For instance, the homogeneous target material content decreases the likelihood of branches formation due to low resistive root for the lightning discharges, while the heterogenous content of the target material increases the least resistance root and generated fulgurites might present several branches in different size. Therefore, there are almost no branches found on our fulgurites, which are generated from homogeneous material. (Fig. 3e,f).

The average length of all fulgurites is measured as nearly 45 mm, except T500—35 mm. It is seen that the length of fulgurites is limited to the specified electrodes’ gap (50 mm). The ratio of the average diameter between the fulgurite (in total nearly 25 mm) and the main central void (around 5 mm) is 5, and it is similar for all the experimental fulgurites, except experiment 500 ms. It is clear that a longer time (continuing current) applied to heat on the target material under the same distance of the electrodes does not change the tube geometry of fulgurite until the density of the molten material is capable of maintaining a unique form during cooling. Once we exceed this point, which is 500 ms in the present study on the particular composition, the tube structure collapses destroying the main voids. On the other hand, we know from other trial experiments that increasing the distance of the electrodes has an effect in reducing the diameter of the central void, regardless of the composition of the target material.

The bulk mass of the fulgurites increases with the duration of the continuing current; however, the density of the fulgurites varies in a very narrow range (around 2.48 ± 0.01 g/cm^3^). That can be explained by the stabilization of the overall volume due to the decrease in porosity (Fig. 5a,b). A longer time of heat transfer (i.e., longer continuing currents) increases the chances for voids to coalesce and eventually collapse, hence reducing the overall porosity. It is thus expected that longer duration experiments would show a bulk density which progressively increases. T300 is the best example for the inversely proportional relationship between porosity and density. In general, the density value of the fulgurites is higher than the pristine LSB (2.42 ± 0.02 g/cm^3^) and the synthesized LSB glass (nearly 2.42 ± 0.004 g/cm^3^). For this particular composition, the pycnometry result show some inconsistencies which could be due to number of reasons: (1) the microporosity of the pristine vesiculated material hindering the repeatability of the density measurements; (2) the limited availability of small fulgurite fragments (< 1 g) with respect to volume of the pycnometer measurement cell; the amount of pristine and partially melted particles of crushed pieces from fulgurites. Nonetheless, the density values of the investigated specimens are very close to each other, therefore we conclude that the variations observed may be considered negligible and within the experimental error.

**Figure 6:**
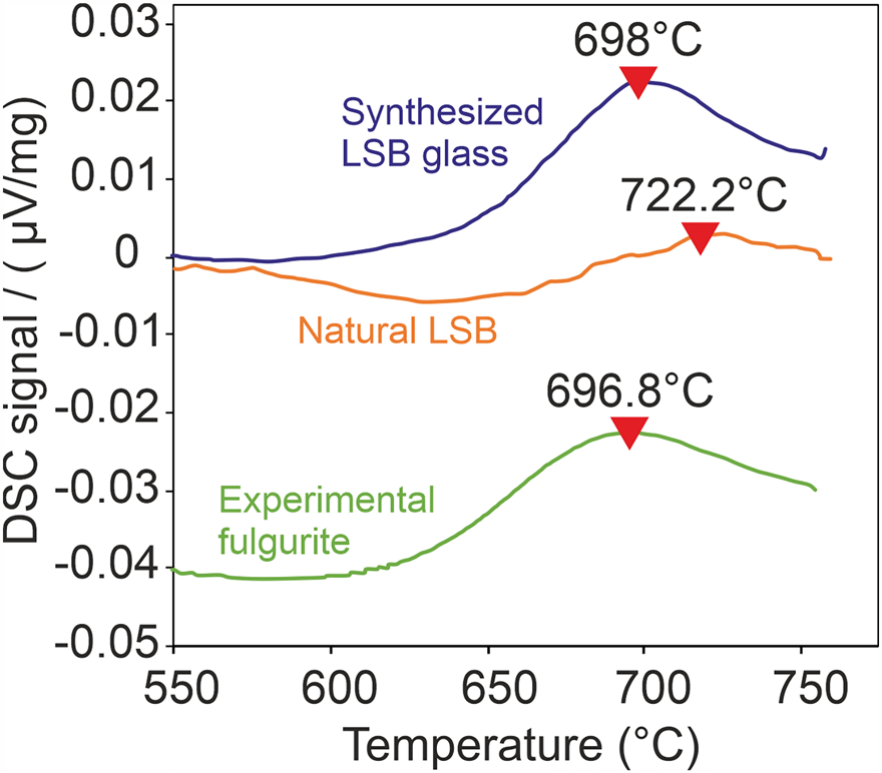
Simultaneous thermal analyses of the experimental fulgurite, synthesized LSB glass and natural LSB specimens (raw data). The revers triangles refers the estimated peak glass transition temperature for each sample.

The dielectric field strength of the target material, directly related to the composition, constrains the current which must be reached by the lightning discharge to kick start the melting. The glass transition temperature (peak) has been determined as ~ 718 °C for the natural LSB sample (glass + crystals), at ~ 694 °C for the experimental fulgurite (glass + possible minor amount of partially melted crystals) and at ~ 693 °C for the remelted LSB glass (Fig. 6). The fulgurite sample and synthesized LSB glass temperatures are identical within the bounds of error (± 1 °C). The glass transition in fulgurite provides a constraint of sample deformation during the lightning strike. The estimated glass transition peak indicates the required temperature for softening the glass of the natural LSB. Despite technical limitations preventing us from determining the possible temperature reached by the plasma, earlier research indicates that it might attain temperatures up to 32,000 K in microseconds^44^. It is noteworthy that 1600 °C represents the lower limit of our simulated arc plasma's minimum temperature, as a thin melt layer coats some residual quartz crystal, which is the most refractory crystal of the pristine mineral assemblage, with a melting point near 1600 °C at atmospheric pressure^45^. The distribution of the glassy and crystalline area in the fulgurites demonstrate also the presence of a notable thermal gradient towards the external surface of all fulgurites. The structure of the grains as a remnant of initial material in the fulgurite might be used relatively to estimate the mean duration of the energy transfer through lightning discharge.

## Conclusion

The experimentally generated fulgurites produced in this study demonstrate a notable similarity with fulgurites found in nature. Thus, our experimental setup and implemented protocol provides a valid pathway that approximately well natural fulgurite formation. Most remarkably, we find that the presence of a continuing current phase in the discharge allows the necessary heat transfer to produce melting of the pristine material.

With these results we can begin to build a calibration for inferring the parameters of the lightning discharges from the characteristics of natural fulgurites. Determination of a minimum temperature of the lightning strike should be possible based on phase melting. The dielectric strength of the target material, directly related to the composition, constrains the current which must be reached by the lightning discharge to generate melting. The forms of the grains as remnant of initial material in the fulgurite can be used to estimate the average duration of the energy transfer via lightning discharge.

Finally, this reconnaissance study of the relationship between lightning and fulgurite formation will hopefully provide an opportunity in the near future to study the significance of this process in generating reactants for prebiotic chemistry on the early Earth.

## Methods

### Sample preparation

To quantify the morphological properties of the fulgurite, all fulgurites were cut perpendicular to their long axis using a precision diamond wire saw. After that half of each fulgurite sample, and around 50 g of natural LSB and synthesized LSB glass were ground in an agate mortar in a vibration mill below to 63 µm for helium pycnometer analysis. Embedded epoxy mounts were also prepared from the other half of each fulgurite for further analysis.

### Scanning electron microscope

The natural apatite and experimentally generated fulgurite were analyzed using a Hitachi SU 5000 scanning electron microscope (SEM) at the Department of Earth and Environmental Sciences of the LMU University of Munich. Backscattered electron images (BSE) of representative portions of the apatite and fulgurite were acquired at varying magnification (i.e., 100×, 200 × ect.). Data collection was conducted by Aztech (version 6.0, Oxford Instrument, AZtechEnergy Advanced EDX-System).

### He-pycnometer analysis

The density of natural LSB, synthesized LSB glass and all fulgurites was measured using a Quantachrome helium pycnometer at LMU University of Munich. Each fulgurite was cut in half longitudinally and these half parts of the fulgurites were used for the measurements. All samples were crushed down to 63 µm to eliminate voids, especially the isolated porosity that can affect the density measurements. Each of the densities were measured at least ten times and the values are presented in Supplementary Table S1.

### Differential scanning calorimetry (DSC)

The calorimetric glass transition temperatures have been measured using a Netzsch 404 C Pegasus. Samples between 50 and 60 mg were placed in a Pt-crucible and heated above the glass transition peak in a dynamic, high purity Argon atmosphere. The samples were than cooled and subsequently reheated with a rate of 10 K/min. Peak glass transition temperatures were extracted from the reheating segments. In Figure 6 the DSC raw curves for the different samples are shown. The samples have been shifted manually for clarity. A temperature correction of − 4.2 °C must be applied to all glass transition temperatures based on a temperature calibration using the melting points of indium, zinc, aluminum, and gold. The error of the temperature measurements is ± 1 °C.

## Supplementary Information

Supplementary Figures.

Supplementary Table 1.

## Data Availability

All data generated or analyzes during this study are included in this published article [and its Supplementary Information files].
