# Supplementary material for: Experimental generation of fulgurite under realistic lightning discharge conditions
Source: Sci Rep. 2023 Jul 19;13:11685. doi: 10.1038/s41598-023-38781-8 (PMC10356921; doi:10.1038/s41598-023-38781-8)
Supplement: Supplementary file 1 — Supplementary Figures. [file 41598_2023_38781_MOESM1_ESM.docx]

**Supplementary Information**

Experimental generation of fulgurite under realistic lightning discharge conditions

A. Zeynep Çalışkanoğlu^1*^, Alessandra S. B. Camara^2^, Corrado Cimarelli^1^, Donald B. Dingwell^1^, Kai-Uwe Hess^1^

^1^Department of Earth and Environmental Sciences, Ludwig-Maximilians-Universität, Theresienstraße 41, 80333 Munich, Germany

^2^Institute of Energy Systems Munich, Universität der Bundeswehr, Werner-Heisenberg-Weg 39, 85577 Neubiberg, Germany

***Corresponding author E-mail address: [zeynep.caliskanoglu@min.uni-muenchen.de](mailto:zeynep.caliskanoglu@min.uni-muenchen.de)


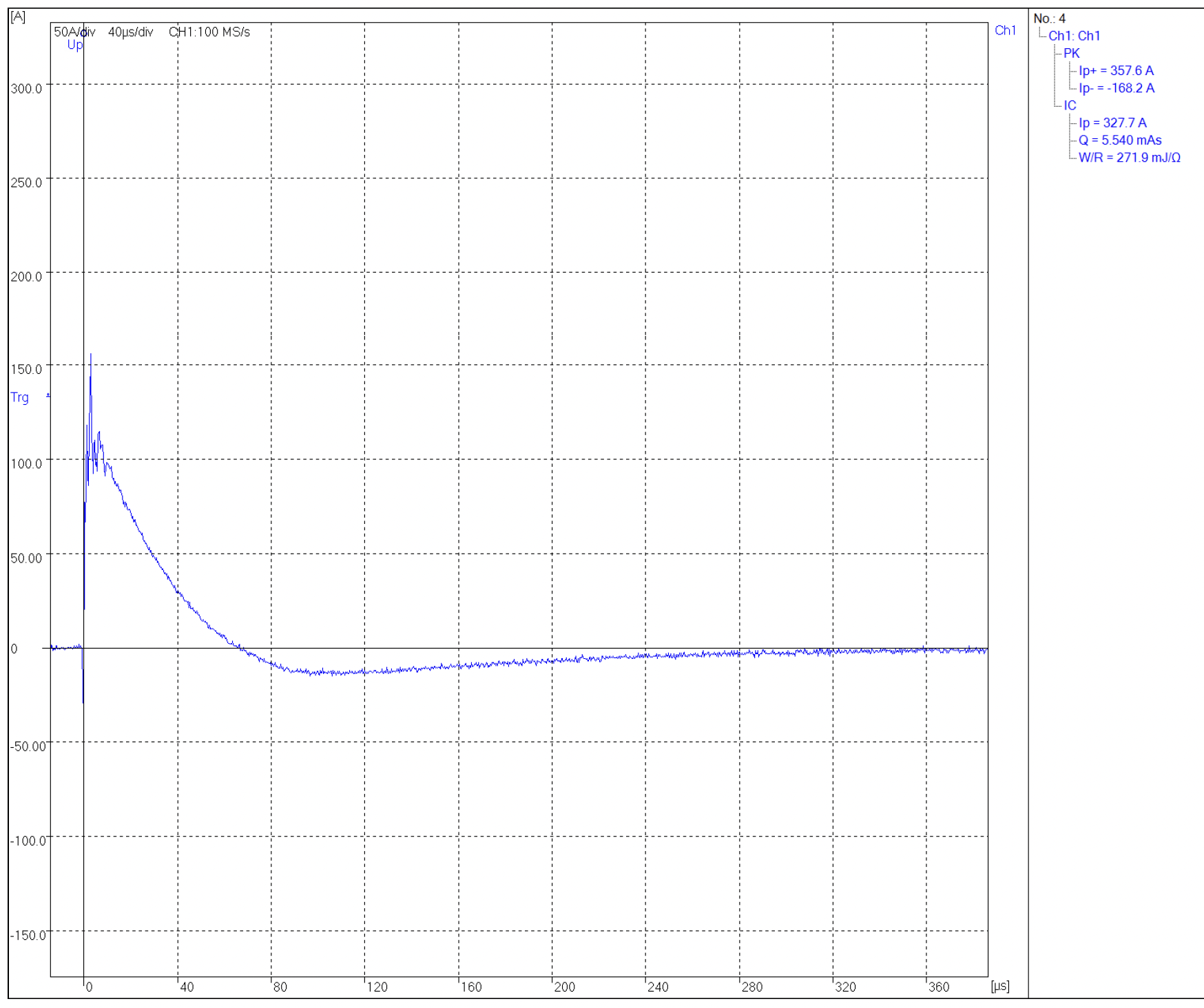


Supplementary Figure S1: Electrical waveform of T0. The target material was exposed only first stroke with no continuing current phase.


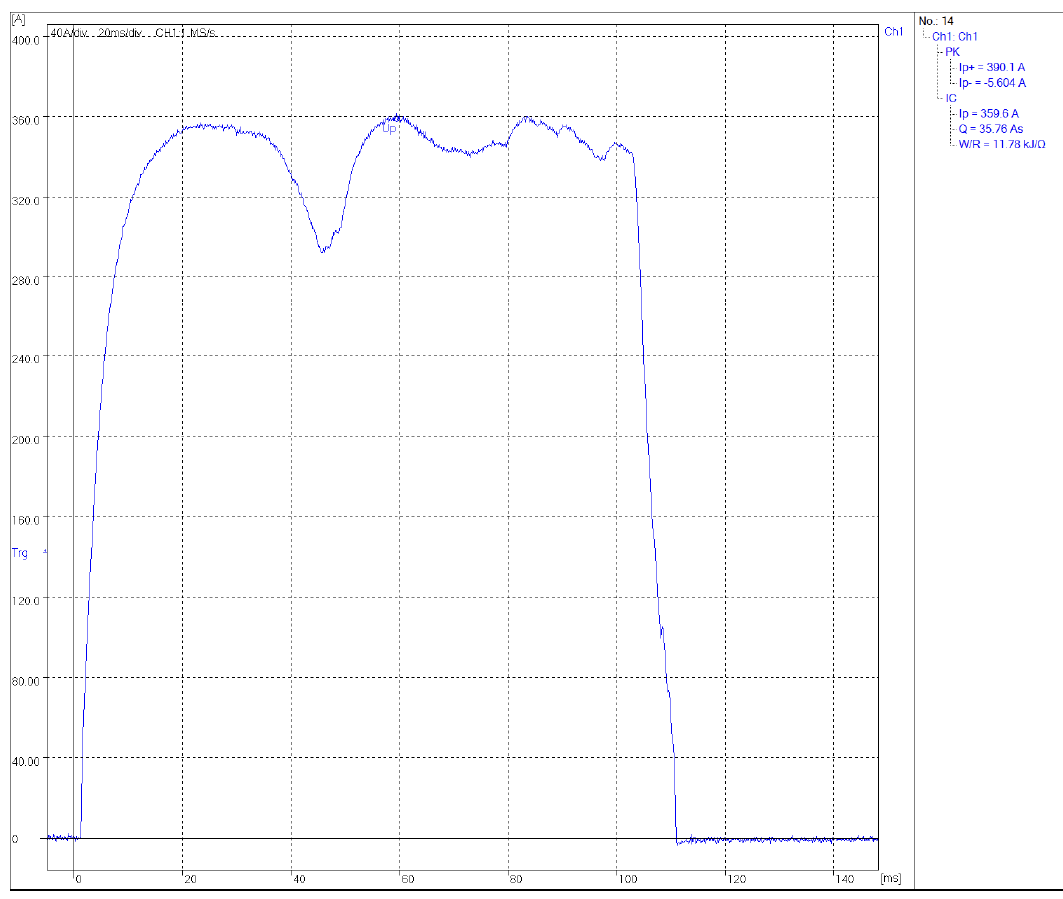


Supplementary Figure S2: Electrical waveform of T100 fulgurite. The experiment was performed by striking the target material with first stroke and continuing current around 100 ms.


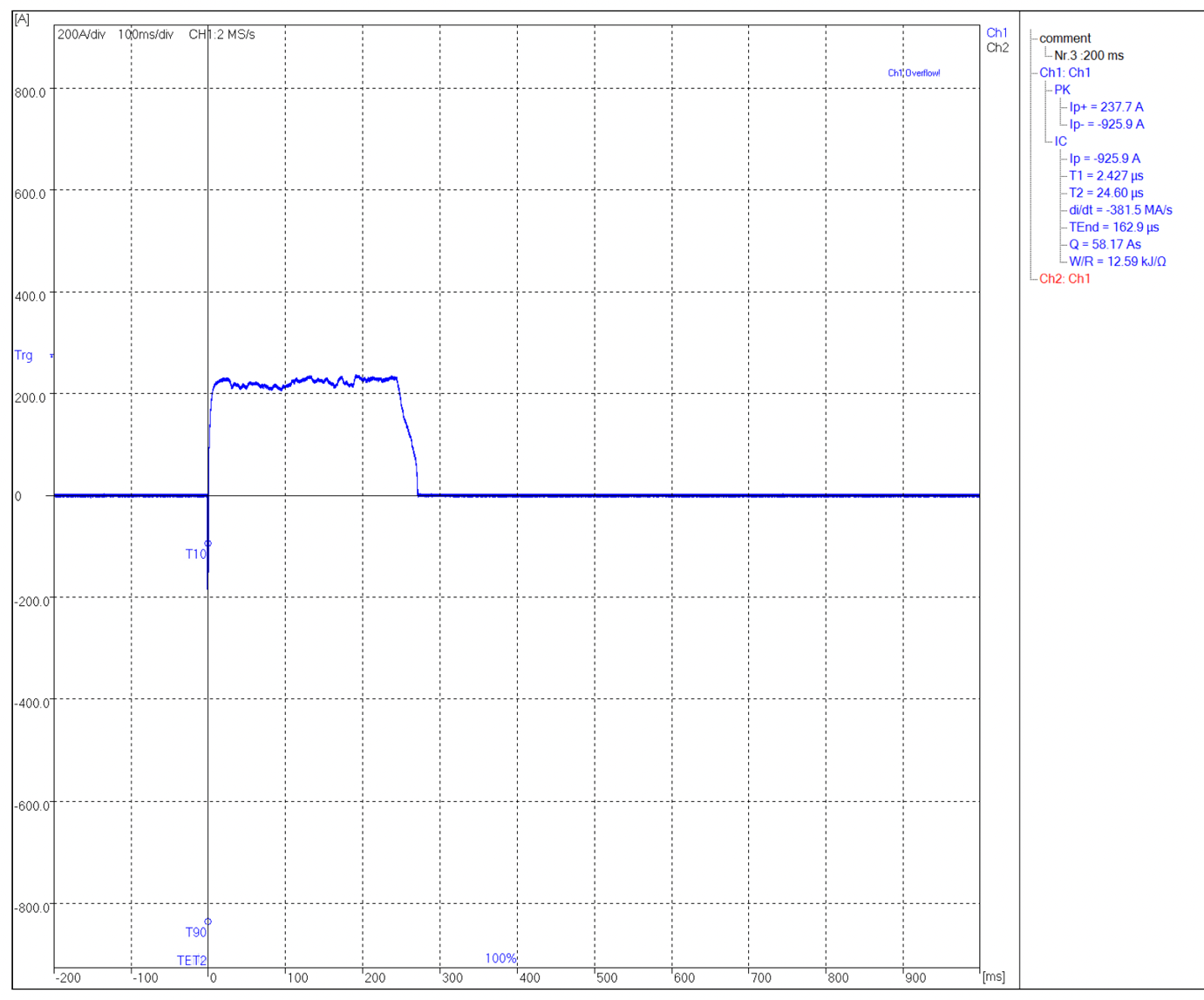


Supplementary Figure S3: Electrical waveform of T200 fulgurite. The experiment consisted of striking the target material with an initial stroke and then maintaining a current for approximately 200 ms.


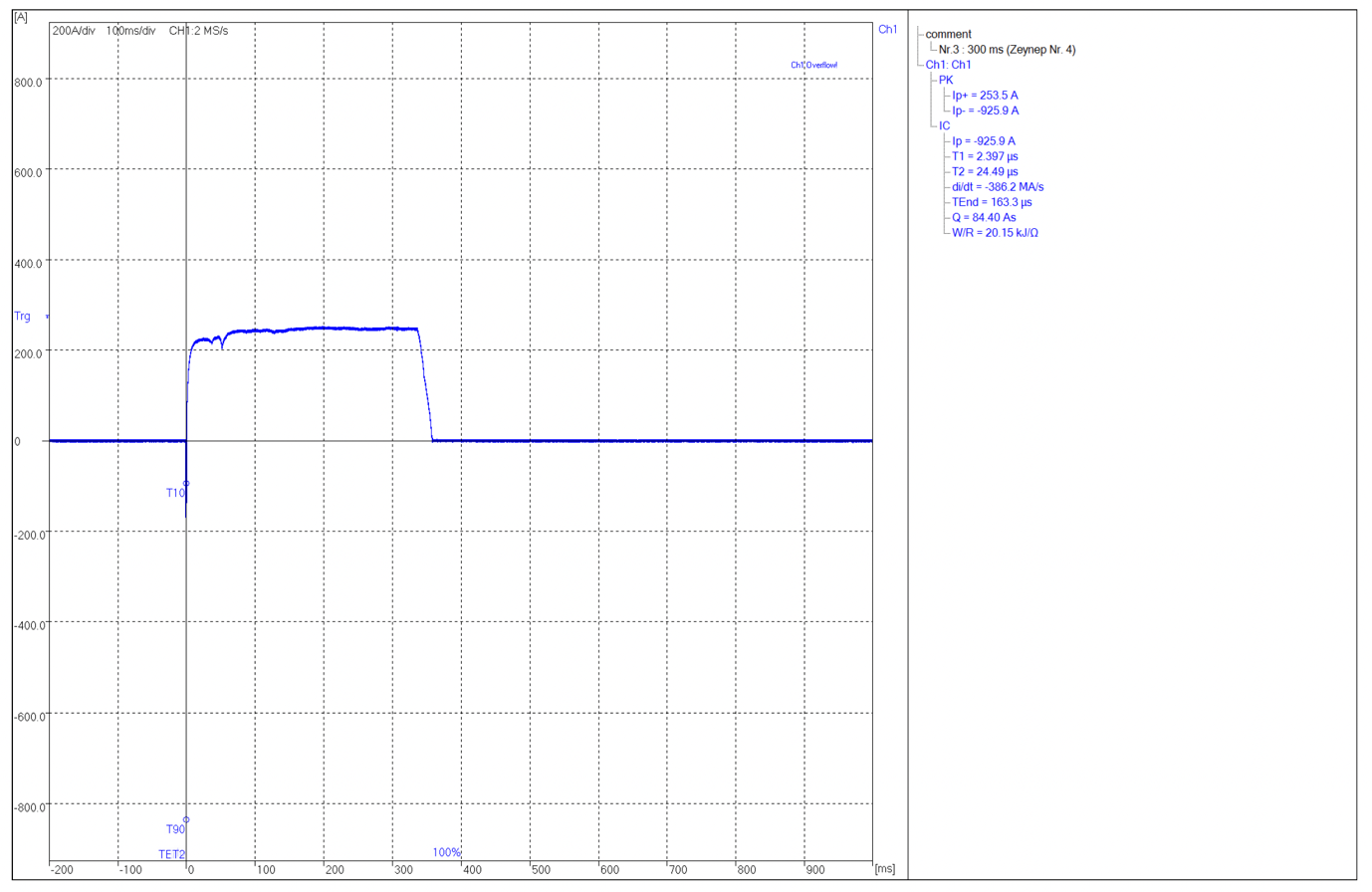


Supplementary Figure S4: Electrical waveform of T300 fulgurite. The experiment was initiated striking the target material with a first stroke, followed by continuing current for approximately 300 ms.

*
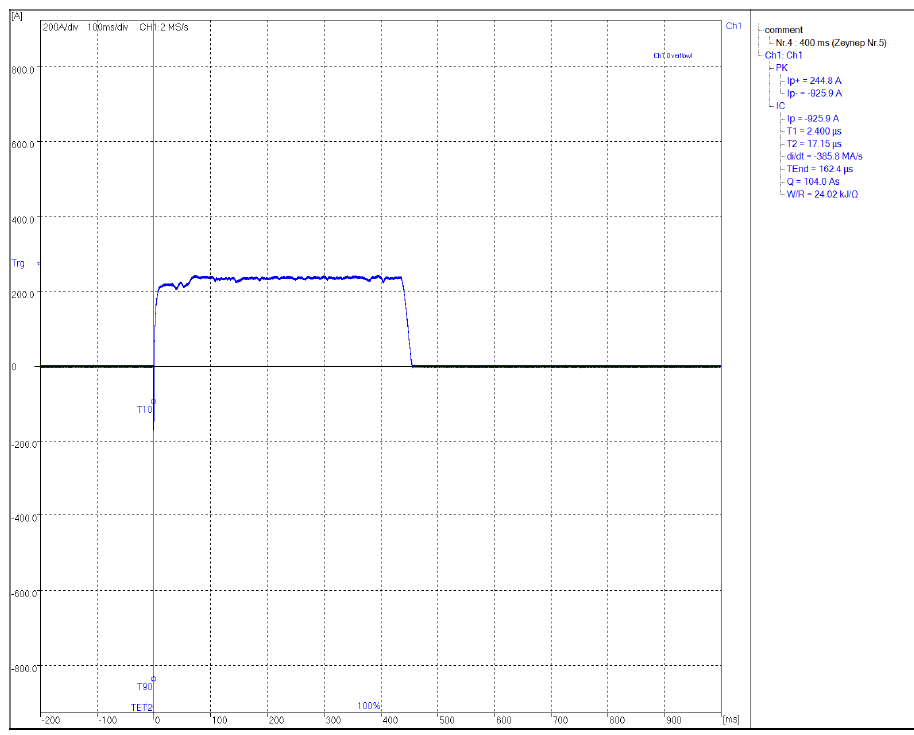
*

Supplementary Figure S5: Electrical waveform of T400 fulgurite. The experiment was performed by striking the target material with first stroke and continuing current around 400 ms.

*
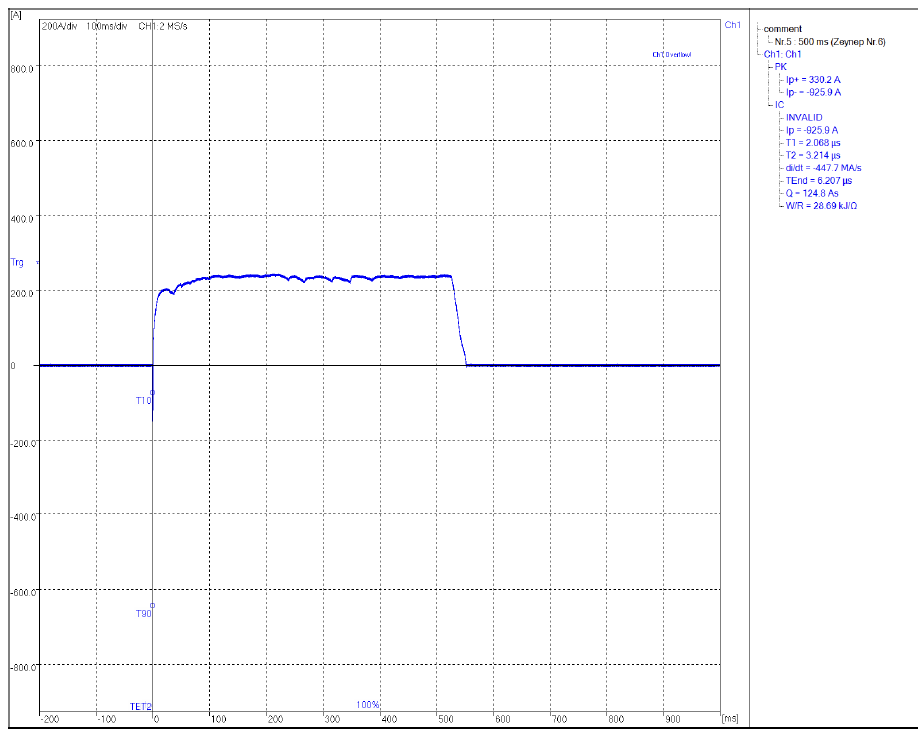
*

Supplementary Figure S6: Electrical waveform of T500 fulgurite. The experiment consisted of striking the target material with an initial stroke and then maintaining a current for approximately 500 ms.
